# CYP2D6 Genotype Dependent Oxycodone Metabolism in Postoperative Patients

**DOI:** 10.1371/journal.pone.0060239

**Published:** 2013-03-28

**Authors:** Ulrike M. Stamer, Lan Zhang, Malte Book, Lutz E. Lehmann, Frank Stuber, Frank Musshoff

**Affiliations:** 1 Department of Anaesthesiology and Pain Medicine, Inselspital, University of Bern, Bern, Switzerland; 2 Department of Anaesthesiology and Intensive Care Medicine, University of Bonn, Bonn, Germany; 3 Department of Forensic Medicine, University of Bonn, Bonn, Germany; Sanjay Gandhi Medical Institute, India

## Abstract

**Background:**

The impact of polymorphic cytochrome P450 CYP2D6 enzyme on oxycodone's metabolism and clinical efficacy is currently being discussed. However, there are only spare data from postoperative settings. The hypothesis of this study is that genotype dependent CYP2D6 activity influences plasma concentrations of oxycodone and its metabolites and impacts analgesic consumption.

**Methods:**

Patients received oxycodone 0.05 mg/kg before emerging from anesthesia and patient-controlled analgesia (PCA) for the subsequent 48 postoperative hours. Blood samples were drawn at 30, 90 and 180 minutes after the initial oxycodone dose. Plasma concentrations of oxycodone and its metabolites oxymorphone, noroxycodone and noroxymorphone were analyzed by liquid chromatography-mass spectrometry with electrospray ionization. CYP2D6 genotyping was performed and 121 patients were allocated to the following genotype groups: PM (poor metabolizer: no functionally active CYP2D6 allele), HZ/IM (heterozygous subjects, intermediate metabolizers with decreased CYP2D6 activity), EM (extensive metabolizers, normal CYP2D6 activity) and UM (ultrarapid metabolizers, increased CYP2D6 activity). Primary endpoint was the genotype dependent metabolite ratio of plasma concentrations oxymorphone/oxycodone. Secondary endpoint was the genotype dependent analgesic consumption with calculation of equianalgesic doses compared to the standard non-CYP dependent opioid piritramide.

**Results:**

Metabolism differed between CYP2D6 genotypes. Mean (95%-CI) oxymophone/oxycodone ratios were 0.10 (0.02/0.19), 0.13 (0.11/0.16), 0.18 (0.16/0.20) and 0.28 (0.07/0.49) in PM, HZ/IM, EM and UM, respectively (p = 0.005). Oxycodone consumption up to the 12^th^ hour was highest in PM (p = 0.005), resulting in lowest equianalgesic doses of piritramide versus oxycodone for PM (1.6 (1.4/1.8); EM and UM 2.2 (2.1/2.3); p<0.001). Pain scores did not differ between genotypes.

**Conclusions:**

In this postoperative setting, the number of functionally active CYP2D6 alleles had an impact on oxycodone metabolism. The genotype also impacted analgesic consumption, thereby causing variation of equianalgesic doses piritramide : oxycodone. Different analgesic needs by genotypes were met by PCA technology in this postoperative cohort.

## Introduction

While morphine represents the standard analgesic in a postoperative setting, other opioids might be also suitable or even advantageous. Oxycodone has been marketed since 1917 and has found widespread use for the treatment of chronic pain, specifically since a controlled-release formula is available. An intravenous formula is now on the market or has been re-launched in several countries. However, intravenous oxycodone is not a standard opioid for postoperative pain management in most countries, including Germany.

As polymorphic cytochrome P450 enzymes (CYP) are involved in the metabolism, a pharmacogenetic impact on oxycodone's efficacy is discussed [1]–[3]. Formation of the active metabolite oxymorphone depends on CYP2D6, whereas *N-*demethylation by CYP3A via the major pathway produces noroxycodone, a metabolite with weak antinociceptive properties. Both metabolites, oxymorphone and noroxycodone, are further degraded to noroxymorphone by CYP2D6 and CYP3A.

Previous experimental trials have demonstrated an impact of CYP2D6 and CYP3A genotypes and enzyme activity on oxycodone's pharmacokinetics, pharmacodynamics and safety in volunteers [2]. In contrast, there are sparse data from postoperative settings and these have not confirmed genotype specific oxycodone consumption and analgesic efficacy [1]. However, surgeries resulted only in minor to medium pain intensities and no difference in opioid consumption could be detected [1]. One might speculate that the impact of genotypes on oxycodone therapy is considerably more profound after major surgeries, which necessitate higher postoperative opioid doses. Thus, possible genotype associated differences in opioid needs may become detectable.

The hypothesis of this study is that oxymorphone plasma concentrations measured during the crucial early postoperative period after major surgeries vary according to CYP2D6 genotypes with an impact on analgesic consumption. In order to translate pharmacogenetic findings into clinical practice, CYP2D6 genotype dependent equianalgesic doses were calculated and compared to piritramide, which is the standard opioid used in Germany. Its metabolism is not dependent on CYP2D6 activity. Thus, equianalgesic intravenous piritramide : oxycodone doses might be helpful for clinicians as there is little experience with oxycodone in a postoperative setting in many countries.

## Methods

### Patients

Approval for this prospective, observational association study was obtained from the institutional review board of the Medical Faculty of the University of Bonn. One-hundred-thirty-one patients scheduled for elective major abdominal surgery or thoracotomy gave written informed consent and were instructed in the details of the study, the use of the patient-controlled analgesia (PCA) device and the numeric rating scale for pain intensities (NRS: 0 denotes no pain, 100 denotes worst pain imaginable). Exclusion criteria were alcoholism, drug dependence, use of CYP3A inducing or inhibiting substances, clinically relevant compromised kidney or liver function, psychiatric diseases, epilepsy, contraindication for the use of study medications, known opioid intolerance, laparoscopic surgery, perioperative epidural analgesia, serious perioperative complications and changes in anesthetic procedure. Preexisting medication was documented and discontinued only for the day of surgery with the exception of drugs necessary for major co-morbidity, e.g. cardiac and pulmonary diseases.

### Clinical Study Protocol

General anesthesia was conducted according to a standardized protocol [1], [4]: propofol 2–3 mg/kg, fentanyl 0.15 mg and cis-atracurium for induction and remifentanil, isoflurane and cis-atracurium for maintenance of anesthesia. About 30 minutes before termination of anesthesia oxycodone 0.05 mg/kg i.v. (oxycodone hydrochloride: Oxygesic® injekt, Mundipharma, Germany) was given with a maximum intraoperative dose of 5 mg. Additionally, dipyrone 1 g i.v. or in case of contraindications to dipyrone acetaminophen 1 g i.v. was infused. Patients' genotypes were unknown during the clinical part of the study.

Prophylactic antiemetic treatment before emergence of surgery was performed in high risk patients for postoperative nausea and vomiting (Apfel's validated risk score ≥3, [5]) according to the department's protocol. After emergence from anesthesia, patients were transferred to the postoperative anesthesia care unit (PACU). The analgesic regimen in the PACU consisted of further doses of oxycodone 1–2 mg if pain scores were >40 at rest. For subsequent analgesic treatment on the general ward, patients could self-administer intravenous bolus doses of 1 ml corresponding to oxycodone 1 mg via a patient-controlled analgesia (PCA) device (Injektomat®-CP PACOM, Fresenius AG, Bad Homburg, Germany) with a lock-out time of eight minutes and no background infusion. If pain management via oxycodone PCA had to be terminated prematurely due to lacking efficacy or side effects, e.g. emesis which could not be controlled by antiemetic medication, the analgesic regimen could be changed to the standard treatment piritramide. Dipyrone 5 g/day and in case of contraindications acetaminophen 4 g/day was infused i.v. as basic non-opioid analgesic regimen in all participants. This is according to the hospital's standard procedure and complies with the national guidelines of a multimodal analgesic regimen [6].

During the 48-hour study period following initial opioid administration, pain scores under rest and exercise/coughing were recorded by the patients using the NRS. Nausea and vomiting (absence or presence) were assessed regularly and treated with antiemetics if needed. Observation time points were hourly up to the eighth hour, at the twelfth hour, and then every six hours up to the forty-eighth hour. Opioid consumption was documented, and the analgesic consumption administered via the PCA device was transferred to an electronic data base. As individual experience and subjective estimation of pain and side effects might differ considerably between investigators and patients, an additional questionnaire was completed by the patients after 48 hours. The questions considered overall patient assessment of pain management and whether the quantity of analgesics was sufficient.

### Genotyping

Blood samples were drawn at 30, 90 and 180 minutes after opioid administration. After centrifugation, blood cells and plasma were frozen separately at -80°C. All laboratory analyses were performed after enrollment of the last patient, and the laboratory staff were blinded to the patients' data.

Genotyping for CYP2D6 *3, *4, *5, *6, *7, *8, *10, *41 and gene duplication/multiduplication as well as for CYP3A5*3 (rs 776746, G6986A) was performed by PCR and real-time PCR as described previously [4], [7]. All alleles with no indicators for one of the genetic variants investigated were categorized as “wild-type” (wt). For translation of the genotypes into a qualitative measure of phenotype, CYP2D6 activity score of each subject was calculated as the sum of the values assigned to each single allele [8]. Alleles *3,*4,*5,*6,*7,*8 were assigned a value of 0, alleles *10,*41 a value of 0.5, the wt allele a value of 1, and wtxN a value of 2 [8]. Four CYP2D6 activity groups were compared: activity score 0 representing poor metabolizers (PM); activity score 0.5–1 representing heterozygous subjects carrying one non-functional allele or a combination of a non-functional with an allele showing reduced function (HZ/IM); activity score of 1.5–2 representing extensive metabolizers or a combination of wild-type allele and reduced function allele (EM); activity score 3 representing ultrarapid metabolizers (UM) with a duplication/multiduplication of a functional allele. Because some authors [2], [9] also suggested a contribution of CYP3A to plasma disposition of metabolites and analgesic efficacy the CYP3A5*3 variant was investigated exploratively. Individuals were assigned to the group of low expressors (CYP3A5*3/*3) or high expressors carrying at least one CYP3A5*1 allele [10].

### Plasma Concentrations of Oxycodone and Its Metabolites

Oxycodone, the metabolites oxymorphone, noroxycodone, and noroxymorphone as well as the deuterated standards oxycodone-d_3_, noroxycodone-d_3_, oxymorphone-d_3_ and hydromorphone-d_3_ were purchased from Cerilliant Corporation (Round Rock, TX, USA). Methanol, water, formic acid (all of HPLC grade), acetonitrile (hypergrade for LC/MS), borate buffer (pH 11), and ammonium formiate were purchased from Merck (Darmstadt, Germany); n-chlorbutane (for HPLC) was obtained from Sigma-Aldrich (Steinheim, Germany).

HPLC mobile phase A consisted of water (HPLC grade) and acetonitrile (90∶10, v/v), mobile phase B consisted of water (HPLC grade) and acetonitrile (10∶90, v/v), both with 0.005 M ammonium formiate and pH was adjusted to 3.5 by addition of formic acid.

Quantification of oxycodone, oxymorphone, noroxycodone and noroxymorphone was performed by a liquid chromatographic-mass spectrometric method with electrospray ionization in positive mode. A previous procedure showed ion suppression effects for the hydrophilic metabolites [11] and was modified: A mixture of 0.2 ml plasma, 20 µl methanolic internal standard solution (1 µg/ml of oxycodone-d_3_, noroxycodone-d_3_, oxymorphone-d_3_ and hydromorphone-d_3_) and 0.1 ml borate buffer were extracted with 1 ml n-chlorbutane. After centrifugation (4000×g, 8 min), the organic phase was transferred and evaporated to dryness under a stream of nitrogen at 60°C. The residue was dissolved in 0.1 ml of mobile phase B and a 10 µl-aliquot was used for chromatography. LC-MS/MS system consisted of a Shimadzu LC 20 series (Duisburg, Germany) high-performance liquid chromatography system (binary pump, degasser, controller and autosampler) coupled with an Applied Biosystems (Darmstadt, Germany) API 4000 QTrap triple quadrupole mass spectrometer. Chromatographic separation was achieved on a Phenomenex (Aschaffenburg, Germany) Hydro RP column (150*2 mm; 4 µm) with a flow of 0.5 ml/min and with following gradients: 0–15 min from 10% to 100% mobile phase B, 15–20 min 100% mobile phase B, 20–21 min from 100% to 10% mobile phase B, 21–25 min equilibration with 10% mobile phase B. For mass spectrometric detection in multiple ion monitoring mode (MRM), following transitions from the molecular ions ([M+H^+^]^+^) were used: oxycodone (316.1→298.0, 241.0), oxycodon-d_3_ (319.1→301.1, 244.1), oxymorphone (302.1→284.0, 227.2), oxymorphone-d_3_ (305.1→287.1, 230.1), noroxycodone (302.2→284.0, 187.0), noroxycodone-d_3_ (305.1→287.0, 230.2), and noroxymorphone (288.1→270.0, 213.0). For quantification, peak area ratios of the analytes to the corresponding deuterated standards were calculated as a function of the concentration of the substances. Noroxymorphone was quantified by referring its peak area to the peak area of oxymorphon-d_3_ due to the lack of its deuterated analogue. The limits of quantification were between 0.08 and 0.11 ng/ml. Precisions and matrix effects were checked according to international guidelines and all criteria were fulfilled [12] The data are summarized in Table 1.

**Table 1:** Limits of detection (LOD; signal-to-noise ratio 3), limits of quantification (LOQ; three times the LOD), precision (at 100 ng/mL), and matrix effects (comparison of analyte responses of post-extraction spiked samples to those of spiked samples).

	LOD ng/ml	LOQ ng/ml	Precision %	Matrix Effect %
Oxycodone	0.02	0.08	8.4	105.6
Oxymorphone	0.03	0.09	9.8	88.8
Noroxycodone	0.03	0.09	11.8	92.5
Noroxymorphone	0.04	0.11	14.9	78.8

### Statistical Analysis

The primary study endpoint was the influence of CYP2D6 genotypes on metabolism and plasma concentrations of oxycodone and its metabolites. As on demand administered drugs *per se* require an alternative approach for analyses of drug and metabolite concentrations due to varying amounts of oxycodone being administered according to individual needs, mean metabolite ratios of oxymorphone/oxycodone plasma concentrations were compared between genotype groups (ANOVA, consecutive post hoc analysis using the Tukey-test). These ratios reflect the CYP2D6 activity-related plasma concentrations of both ocycodone and oxymorphone at the time points for blood sampling [1]. From previous data it was conservatively assumed that in PM this ratio was about one third of that in EM and UM with a standard deviation being as high as the means of PM [1], [13]. For CYP2D6 7–10% of Caucasian individuals are PM. A total minimum number of at least 120 patients was calculated to provide sufficient pharmacokinetic data for the PM group. The x^2^-goodness of fit test was applied to all SNPs to ascertain whether they were in Hardy-Weinberg equilibrium.

As a secondary endpoint, a comparison of genotype-dependent cumulative analgesic consumption that measured titration doses in the recovery room as well as delivered PCA bolus doses was performed (repeated measures ANOVA). For analysis of equianalgesic doses, a comparison to a cohort receiving the standard treatment with piritramide, a synthetic opioid structurally related to meperidine, was used. Depending on their body weight, patients received piritramide 4–8 mg i.v. before the end of surgery. In the PACU, further doses of piritramide 2–3 mg were titrated if pain scores were >40 at rest. The setting used on the PCA device was identical to the oxycodone group, however, the 1 ml bolus dose consisted of piritramide 2 mg. This ratio was chosen due to a lower relative analgesic potency of piritramide compared to morphine [14], [15].

Equianalgesic ratios piritramide : oxycodone were calculated from piritramide doses versus oxycodone doses titrated in the recovery room and from the delivered amount of the respective opioid via PCA. For the opioid consumption via PCA the first eight hours were represented in hourly intervals. Thereafter, opioid consumption up to the 12^th^, 18^th^, 24^th^, 30^th^, 36^th^, 42^nd^ and 48^th^ hour (15 observation time points) was extracted from electronic PCA protocols. Patients were allocated to genotype dependent CYP2D6 activity groups with no (PM), one (HZ/IM) or at least two active CYP2D6 alleles (EM+UM). Mean overall equianalgesic ratios piritramide : oxycodone with standard deviations (SD) and 95%-confidence intervals (95%-CI) were calculated and compared by ANOVA followed by post-hoc analysis.

For all analyses level of significance was defined as p<0.05 with subsequent correction for multiple testing. Analyses were performed by using the statistical software STATISTICA 10 (Stat Soft, Inc. Tulsa, OK, USA).

## Results

### Demographic Data and Genotypes

In this trial, a total of 131 patients were enrolled. Complete data for 121 patients on oxycodone could be analyzed (major urologic surgery: 80 patients, major abdominal: 29, liver/pancreatic surgery: 5, thoracotomy 4, major gynecological laparotomy: 3 patients). Ten patients had to be excluded due to violation of the study protocol, need for prolonged postoperative mechanical ventilation or surgical complications. Demographic and surgery-related as well as frequency of genotype groups are displayed in Table 2.

**Table 2:** Demographic and Perioperative Data.

	PM^a^	HZ/IM^a^	EM^a^	UM^a^
Number of patients (%)	8 (6.6)	38 (31.4)	70 (57.9)	5 (4.1)
Male/female	5/3	21/17	48/22	3/2
Age (years)	63±9	58±13	56±15	50±12
Body weight (kg)	82±16	81±33	86±30	88±20
Height (cm)	177±10	165±24	167±32	176±3
Duration of surgery (min)	192±45	200±82	203±91	193±73
ASA I/II/III/IV^b^	1/6/1/0	5/22/10/1	11/36/22/1	1/2/2/0
CYP3A5 *1/*1 or *1/*3 or *3/*3	0/0/8	0/4/34	0/9/62	0/0/5

Patients were clustered according to CYP2D6 genotypes. Measures represent mean±SD or number (%) of patients.

a *: PM = poor metabolizers, HZ/IM = heterozygous subjects or intermediate metabolizers, EM = extensive metabolizers, UM = ultrarapid metabolizers.*

b *: ASA = American Society of Anesthesiologists physical status; I = healthy patient, II = mild systemic disease, III = severe systemic disease, IV = severe systemic disease that is a constant threat to life.*

### CYP2D6 and CYP3A Genotypes

Of the participants enrolled, 104 were of German descent, the remainder from other European countries (13), Arabia or Africa (4). Genotyping was successful in all blood samples. The observed CYP2D6 allele frequencies were 2.0% (95%-CI: 0.9/4.7) for *3, 17.4% (13.1/22.6) for *4, 2.9% (1.4/5.9) for *5, 0.6% (0.4/3.6) for *6, 4.5% (2.6/7.9) for *10, 7.9% (5.1/11.9) for *41 and 2.1% (0.9/4.7) for wtxN. The CYP2D6*7 allele was not detected. For CYP3A5, the allele frequency of *3 was 94.1% (91.7/95.9). Allele frequencies did not differ when considering subjects of European descent only. There was no deviation of allele frequencies from Hardy-Weinberg equilibrium (p-values >0.05) and results were in agreement with data reported previously [16], [17]. Considering genotype dependent metabolic activity 6.6% and 4.1% of the patients carried a CYP2D6 dependent activity score of 0 or 3 (Table 2). For CYP3A, 10.7% high expressors with at least one wt-allele were detected (Table 2).

### Genotype-Dependent Plasma Concentrations

**Figure 1:**
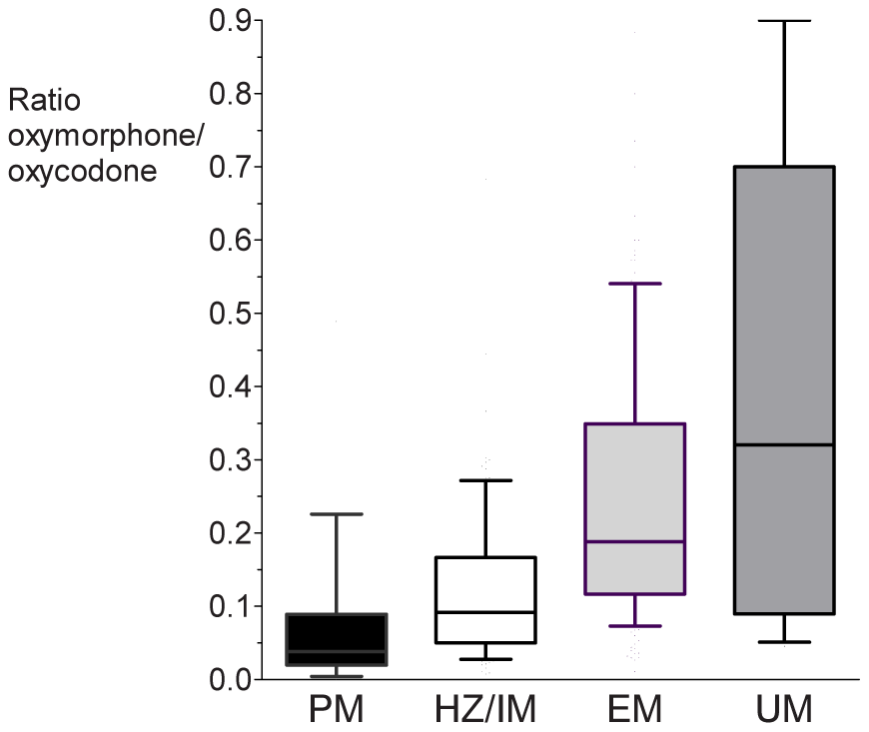
Mean Ratio of Oxymorphone/Oxycodone Plasma Concentrations depending on CYP2D6 Genotype Groups. Boxes represent 1^st^ and 3^rd^ quartile; whiskers the 5^th^ and 95^th^ percentiles. ANOVA p = 0.001; Tukey-test: PM vs. UM: p = 0.009, HZ/IM vs. UM: p = 0.005).

**Figure 2:**
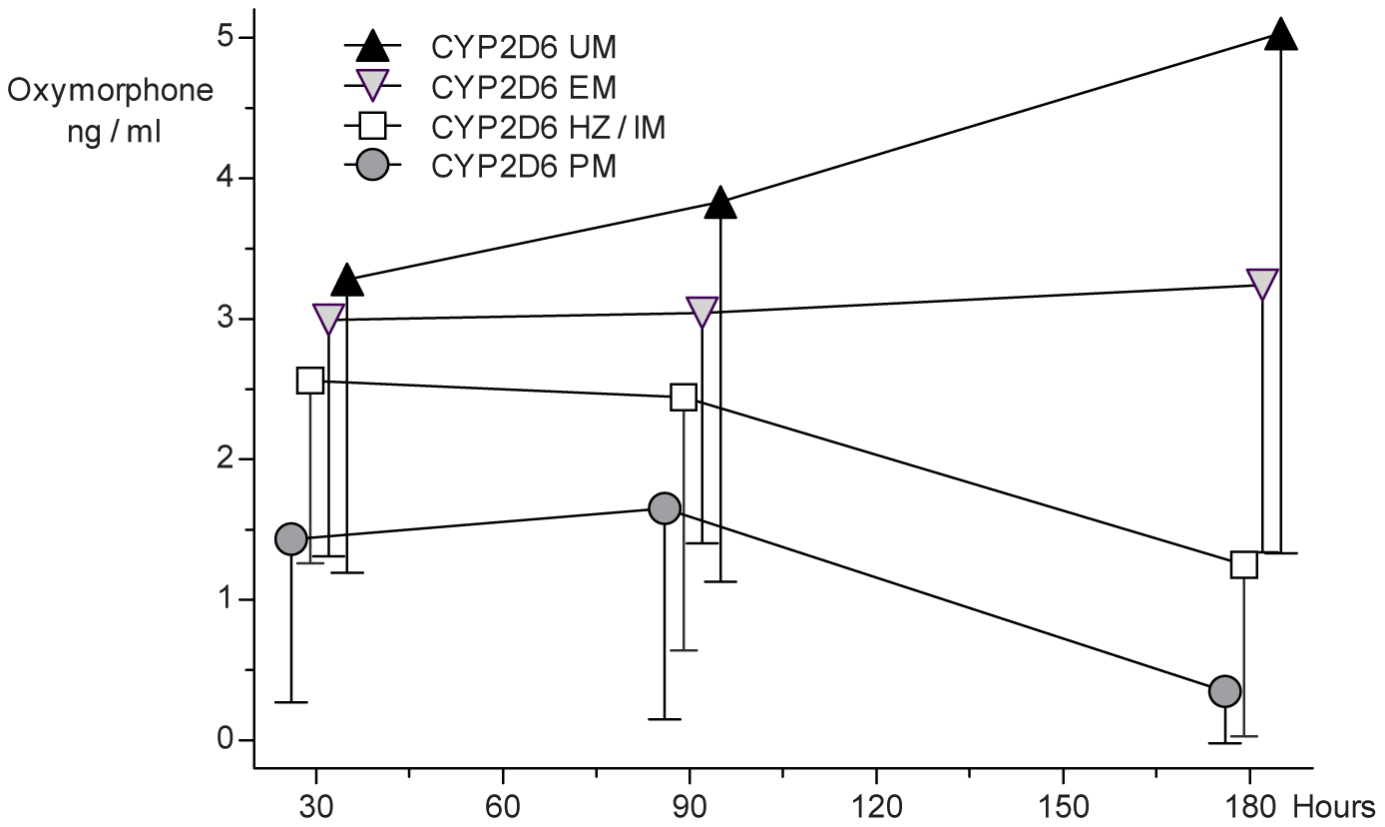
Plasma Concentrations of Oxymorphone. Oxymorphone concentrations were clustered to CYP2D6 genotype activity groups PM, HZ/IM, EM and UM. Measures represent means with -SD. Comparison of genotype groups by repeated measures ANOVA, p = 0.004.

Plasma concentrations of oxycodone and oxymorphone were dependent on CYP2D6 activity groups. The resulting metabolite ratio oxymorphone : oxycodone was lowest in PM and highest in UM (p = 0.001; Figure 1). The time course of plasma concentrations showed lowest oxymorphone doses in PMs (comparison of genotype groups by repeated measures ANOVA, p = 0.004; Figure 2). There was no difference in plasma concentrations for noroxymorphone among CYP2D6 genotypes (concentrations at 30 minutes: PM 2.8±3.2, HZ/IM 2.2±1.7, EM 3.4±2.5, UM 2.9±2.5 ng/ml; p = 0.9). For CYP3A5*3/*3 carriers neither plasma concentrations of oxycodone (p = 0.5), nor the concentrations of noroxycodone (p = 0.4) or noroxymorphone (p = 0.8) differed compared to those subjects carrying at least one wt-allele.

### Analgesic Consumption and Efficacy

**Figure 3:**
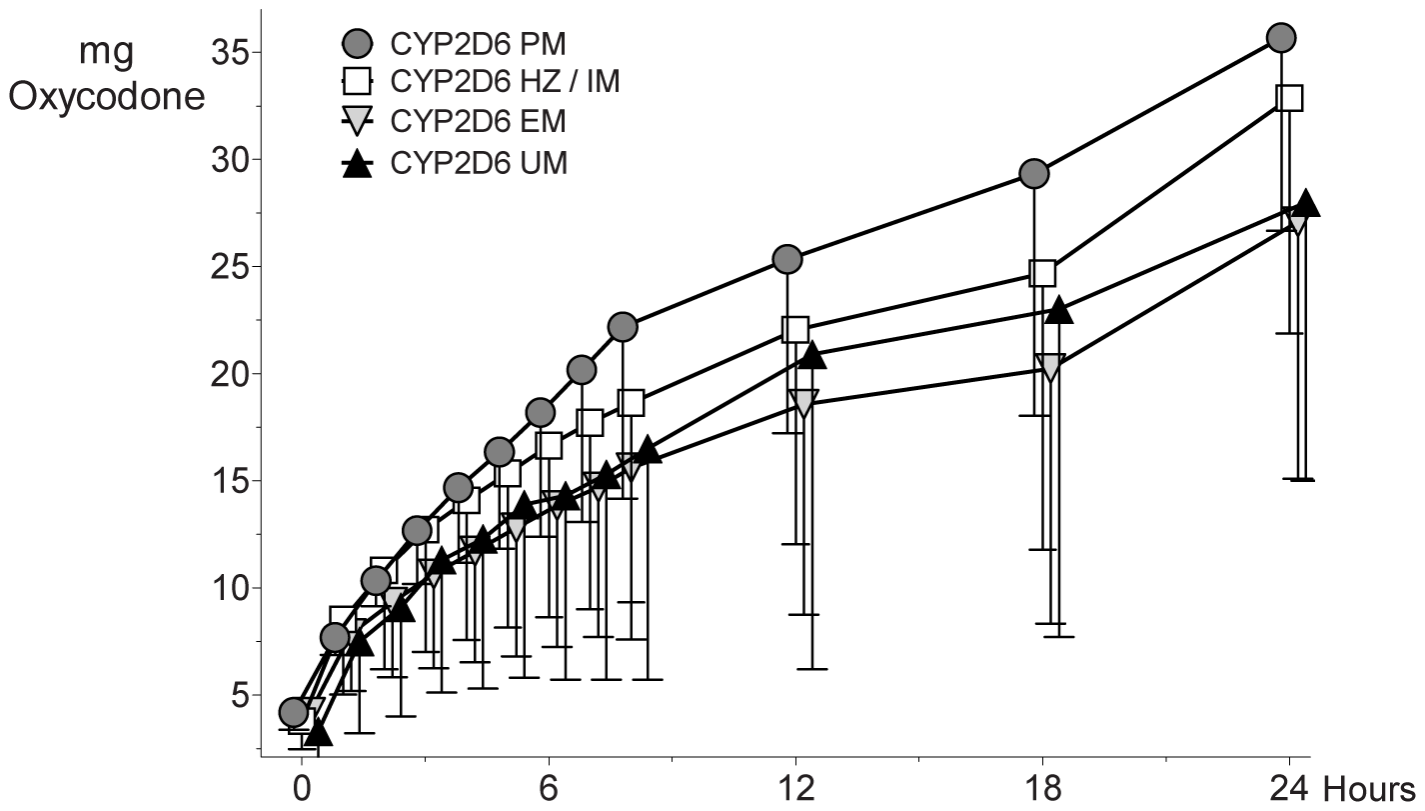
Cumulative Oxycodone Consumption. Patients were allocated to CYP2D6 genotype groups. Data are presented as mean with -SD. Repeated measures ANOVA, p = 0.005 for consumption up to the 12^th^ hour. Thereafter, there was no significant difference after correction for multiple testing.

Sixty patients (PM: 3, HZ/IM: 24, EM: 32, UM: 1) needed an additional oxycodone dose (3.3±4.3 mg) in the recovery room (no difference between CYP2D6 genotypes). The cumulative oxycodone consumption up to the twelfth hour varied between the CYP2D6 activity groups (Figure 3, repeated measures ANOVA, p = 0.005; post-hoc analysis PM versus EM: p<0.001; PM versus carriers of at leat one active allele: p = 0.002). For CYP3A activity groups, no difference in analgesic consumption could be detected (CYP3A high ecxpressors 20.7±9.6 mg oxycodone up to the twelfth hour, CYP3A low expressors: 20.0±10.5 mg). Pain scores at rest and movement did not differ between CYP2D6 genoytpye groups (Table 3) and none of the patients had to be switched to analgesic rescue medication. The postoperative questionnaire revealed a high-degree of patient satisfaction. Only two individuals on oxycodone (PM:1, EM:1) judged pain management as insufficient. Twelve patients (PM: 2, HZ/IM:4, EM:6, UM:0) answered “no” to the question whether delivered opioid doses were high enough.

**Table 3:** Pain Scores at Rest and Coughing/Movement.

		Oxycodone			Piritramide
	PM^a^	HZ/IM^a^	EM^a^	UM^a^	
**On arrival at the PACU** b	40 (30/50)	43 (30/51)	30 (15/45)	27 (20/62)	40 (30/50)
**2 hrs at rest**	40 (30/40)	40 (30/45)	30 (20/40)	27 (20/37)	36 (30/44)
**2 hrs at coughing**	65 (60/70)	60 (50/70)	57 (50/70)	70 (50/80)	60 (50/70)
**6 hrs at rest**	27.5 (20/39)	30 (22/38)	26.6 (20/35)	25 (27/30)	30 (20/37)
**6 hrs at coughing**	55 (50/60)	57.5 (50/70)	50 (41/60)	70 (60/70)	50 (40/60)
**12 hrs at rest**	24 (16/30)	30 (20/33)	25 (15/30)	30 (30/30)	20 (11/30)
**12 hrs at coughing**	50 (48/60)	52.5 (50/60)	50 (40/60)	60 (60/70)	40 (39/50)

Patients treated with oxycodone were clustered according to CYP2D6 genotype groups. Additionally, the results of the piritramide groups are displayed. Pain scores are presented as medians (1^st^/3^rd^ quartile).

a *: PM = poor metabolizers, HZ/IM = heterozygous subjects or intermediate metabolizers, EM = extensive metabolizers, UM = ultrarapid metabolizers.*

b : PACU = post anesthesia care unit.

For comparison of equianalgesic doses, an additional cohort of 125 patients on piritramide were analyzed. Demographic and sugery-related data as well as genotypes and pain scores were comparable to the oxycodone group (Tables 2–4). Equianalgesic doses of piritramide versus oxycodone differed between CYP2D6 genotypes (Table 5). For the combined group of EM and UM this ratio was higher compared to PM and HZ/IM (p<0.001).

**Table 4:** Demographic and Perioperative Data of 125 Patients receiving Piritramide.

	Piritramide
**Male/Female**	69/56
**Age** (years)	56±15
**Body weight** (kg)	79±20
**Height** (cm)	170±17
**Duration of surgery** (min)	210±78
**ASA I/II/III/IV** a (no. patients)	18/71/33/3
**Kind of surgery** (no. patients)	
Urologic (nephrectomy/enucleation of kidney tumor, prostatectomy)	81
Major abdominal (bowel resection)	32
Liver/pancreatic surgery	4
Thoracotomy (lung resection, thymectomy)	6
Major gynecological laparotomy	2
**CYP2D6 genotype group:** PM/HZ/IM/EM/UM^b^	13/39/69/4
**CYP3A5** *1/*1 or *1/*3 or *3/*3	3/10/112

Data represent number of patients and means±SD.

a : *ASA: American Society of Anesthesiologists physical status;* I = healthy patient, II = mild systemic disease, III = severe systemic disease, IV = severe systemic disease that is a constant threat to life.

b *: PM = poor metabolizers, HZ/IM = heterozygous subjects or intermediate metabolizers, EM = extensive metabolizers, UM = ultrarapid metabolizers.*

**Table 5:** Equianalgesic Doses (Ratio Piritramide versus Oxycodone Consumption).

	PM^a^	HZ/IM^a^	EM+UM^a^
**Mean**	1.61	1.72	2.17
**SD**	0.32	0.20	0.22
**95%-CI**	1.43/1.77	1.61/1.83	2.05/2.29^b^

Patients receiving oxycodone were allocated to genotype-dependent CYP2D6 activity groups PM, HZ/IM, and EM+UM. Mean ratios with standard deviations (SD) and 95%-confidence intervals (95%-CI) were calculated from the mg doses titrated in the recovery room, the delivered mg via PCA during the first 8 hours, and the delivered mg via PCA up to the 12^th^, 18^th^, 24^th^, 30^th^, 36^th^, 42^nd^ and 48^th^ hour.

a *: PM = poor metabolizers, HZ/IM = heterozygous subjects or intermediate metabolizers, EM = extensive metabolizers, UM = ultrarapid metabolizers.*

b : ANOVA p<0.001, posthoc analysis PM versus EM+UM and HZ/IM versus EM+UM: p<0.001.

## Discussion

In patients receiving oxycodone for postoperative analgesia after major surgery, the CYP2D6 genotype influenced the ratio of plasma concentrations of oxymorphone/oxycodone as well as analgesic consumption via PCA during the first 12 postoperative hours. This confirms our hypothesis and demonstrates that sufficiently high pain scores resulting in relevant analgesic needs are necessary to detect genotype dependent differences.

### Influences of CYP2D6 Genotypes on Plasma Concentrations

The CYP2D6-dependent metabolite oxymorphone has a 40 to 45-fold higher μ-opioid receptor binding affinity than oxycodone [18]–[21] and has proved to be a more potent μ-opioid receptor agonist. However, its impact on analgesia is controversial since formation of oxymorphone is not considered the major metabolic pathway [18], [22].

In a previous trial, plasma concentrations were measured 25 minutes after i.v. injection of oxycodone 5 mg (PM: 0.04 ng/ml; EM: 0.12 ng/ml) [1]. Similar to the present findings oxymorphone concentrations and the metabolite ratio oxymorphone ∶ oxycodone varied depending on CYP2D6 genotypes, but overall substance concentrations measured in plasma were lower than in the present study, which might be due to different laboratory techniques [1]. Furthermore, another panel of SNPs was investigated resulting in a different classification of CYP2D6 activity status [1].

A decrease of oxymorphone concentrations and a shift to the N-demethylation pathway was reported in subjects with blocked CYP2D6 activity (by comedication with quinidine), which resembles the PM status [23]. In 20 chronic-pain patients, co-administration of paroxetine decreased plasma AUC of oxymorphone by 67% and increased AUC of noroxycodone by 100%, but had no effect on oxycodone analgesia or the use of rescue medication [22]. In contrast, the effect of paroxetine on plasma concentrations of one single i.v. dose of oxycodone was negligible in an experimental setting [3]. In these previous investigations, either no genotyping was performed or no sufficient number of subjects was enrolled to perform a genetic association study.

### CYP3A Activity

The CYP3A4 pathway is described as quantitatively more important [18] with the N-demethylated metabolite noroxycodone showing poor antinociceptive effects [19], [24], [25]. CYP3A is the most abundant CYP protein in the human liver, and the influence of genetic variants on metabolism has been demonstrated in immunosuppressive drugs with a narrow therapeutic index and frequent side effects [26]–[28]. In contrast, data on other widely used drugs metabolized by CYP3A are sparse. For healthy volunteers, Samer and co-workers stated that oxycodone's pharmacokinetics is also modulated by CYP3A activity [29]. A higher noroxycodone/oxycodone ratio and a higher daily oxycodone escalation rate was described in cancer patients carrying the CYP3A5*3/*3 genotype [9], however, an association of plasma concentrations and analgesic consumption to CYP3A5 genotype could not be confirmed in the present trial. It is well described that comedication with voriconazole, itraconazole, telithromycin, rifampin or ketoconazol produces considerable changes in oxycodone's pharmacokinetic [2], [3], [29]–[33] and even foods like grapefruit juice can inhibit CYP3A activity with respective interactions [34]. Several authors have pointed out that dose adjustment of oxycodone might be necessary, when used concomitantly with CYP inducers or inhibitors to either maintain adequate analgesia or prevent overdosing [31], [32]. However, data from large-scale clinical studies are lacking thus far.

### Genotype and Opioid Consumption

The central nervous system effects of oxycodone were described as governed by the parent drug, with a negligible contribution from its oxidative and reductive metabolites [18], [22]. This hypothesis was mainly based on the low contribution of CYP2D6 to the overall metabolism of this opioid [18], [22], however, this hypothesis was not confirmed in all human trials. Specifically in some volunteer studies, oxymorphone did play a role for analgesic efficacy in parallel to the clear-cut pharmacokinetic effects described in nearly all publications in which this issue has been addressed [1], [13], [29].

In experimental pain models enrolling a limited number of volunteers, oxycodone analgesia was reduced in PM compared to EM, whereas increased pharmacodynamic effects were described in two UM [13], [29]. Some case reports [35]–[38] as well as the present results are in line with these findings. In contrast, no genotype dependent difference in analgesic consumption was detected in a previous PCA study [1], but, no differentiation of UM and HZ/IM was performed, surgical procedures were less invasive and the 24 h oxycodone consumption was considerably lower with about 40% of the patients not using the PCA device at all [1]. The overall low analgesic needs might have masked possible differences between genotypes. Stubhaug and co-workers stated that a sufficiently strong base-line pain is necessary to discriminate between drugs [39] or as in this case between different genotypes. As hypothesized in the present trial, enrolling patients undergoing major surgery PM needed more oxycodone. A substantial change in the analgesic regimen for postoperative PCA seems not to be necessary as PM could compensate higher analgesic needs by demanding additional PCA bolus doses and titrating themselves to comfortable low pain intensities. This is also reflected by comparable pain scores in the different CYP2D6 activity groups.

The definition of equianalgesic doses of oxycodone to morphine has been described as difficult due to pharmacokinetic differences of the drugs [40]. For morphine∶oxycodone a ratio of about 1.5 has been suggested [41]–[43]. In a further trial reporting a ratio of 1.0 in patients undergoing non-abdominal surgeries, high PCA bolus doses (oxymorphone 30 µg/kg) might have contributed to an increased overall opioid consumption [44]. Thus, possible differences in opioid potency may have been concealed.

Equianalgesic doses of piritramide∶oxycodone have not been reported up to now. They are useful for clinicians in the case of opioid switching. Piritramide is the preferred opioid in a postoperative setting in several European countries due to rapid onset, absence of active metabolites, and unproblematic use, also in the case of impaired renal function [14], [15]. Due to higher oxycodone consumption in PM, the present trial revealed a respective change in equianalgesic dose ratios piritramide∶oxycodone compared to subjects carrying at least two wild-type alleles.

There are some limitations in the current study. First, the overall number of patients included in this trial is limited. Nevertheless, the results show a significant association between CYP2D6 genotypes and oxycodone metabolism and consumption at a statistical power of 80%. Second, for analysis of equianalgesic doses piritramide∶oxycodone a double-blinded study design might have been suitable as well. However, as the patients' genotypes were unknown during the clinical part of the trial and the drugs were administered via PCA by the patients themselves, the influence of physicians and nurses on analgesic consumption should be negligible. For more detailed evaluation of genotype-associated oxycodone effects and side effects a larger patient cohort needs to be investigated in a future trial. Additionally, the influence of concomitant medication interfering with CYP activity has to be addressed in a postoperative setting.

## Conclusions

In this patient cohort recovering from major surgery and requiring clinically relevant opioid doses, a CYP2D6 genotype-dependent effect on plasma concentrations of oxycodone and oxymorphone was detected. The higher oxycodone consumption in PM resulted in genotype specific equianalgesic doses of piritramide∶oxycodone. PCA technology overcomes differences in doses needed by various genotype groups, so that the PM also experienced sufficient pain relief from oxycodone in this postoperative setting.
